# Candidate New Species of *Kobuvirus* in Porcine Hosts

**DOI:** 10.3201/eid1412.080797

**Published:** 2008-12

**Authors:** Gábor Reuter, Ákos Boldizsár, István Kiss, Péter Pankovics

**Affiliations:** ÁNTSZ Regional Institute of State Public Health Service, Pécs, Hungary (G. Reuter, Á. Boldizsár, P. Pankovics); Veterinary Diagnostic Directorate, Debrecen, Hungary (I. Kiss)

**Keywords:** Picornavirus, kobuvirus, swine, porcine kobuvirus, letter

**To the Editor**: Picornaviruses (family *Picornaviridae*) are small, nonenveloped viruses with single-stranded, positive-sense genomic RNA, currently divided into 8 genera: *Enterovirus*, *Aphthovirus*, *Cardiovirus*, *Hepatovirus*, *Parechovirus*, *Erbovirus*, *Teschovirus*, and *Kobuvirus* (*1*). To date, the genus *Kobuvirus* consists of 2 species, *Aichi virus* and *Bovine kobuvirus,* each possessing 1 serotype. Aichi virus (strain A846/88) was first isolated from a stool sample obtained from a person with acute gastroenteritis in 1991 (*2*). Bovine kobuvirus (strain U-1) was detected in bovine sera and in feces samples from clinically healthy cattle in 2003 (*3*). Human and bovine kobuviruses were first isolated in Japan. Recently, kobuviruses have also been detected in humans in other countries in Asia (*4*), Europe (*5**,**6*), and South America (*5*) and in calves with diarrhea in Thailand (*7*). The Aichi virus and bovine kobuvirus genomes are approximately 8.2–8.3 kb, respectively, and both have a typical picornavirus genome organization, including the L protein following structural (VP0, VP3, and VP1) and nonstructural (2A–2C and 3A–3D) regions (*1**,**3*). Genetic identity between Aichi and U-1 viruses ranges from 47.7% (3′ untranslated region) through 70.8% (3D region) (*3*). In this study, we report a new candidate species of kobuvirus. Porcine kobuvirus was serendipitously detected in fecal samples from domestic pigs in Hungary.

Fecal samples were collected in February 2007 from 15 healthy piglets (*Sus scrofa domestica*) <10 days of age from a farm in Ebes located in eastern Hungary. The aim of the study was to detect porcine calicivirus (norovirus and sapovirus) in domestic pigs by using reverse transcription–PCR (RT-PCR), using the generic primer pairs p289/p290 designed for the calicivirus RNA-dependent RNA polymerase gene (319 nt for norovirus or 331 nt for sapovirus) (*8*). RNA isolation and RT-PCR were performed as described previously (*9*). PCR products were sequenced directly in both directions with the BigDye Terminator Cycle Sequencing Ready Reaction Kit (PE Applied Biosystems, Warrington, UK) by using the PCR primers and run on an automated sequencer (ABI PRISM 310 Genetic Analyzer; Applied Biosystems, Stafford, TX, USA). Phylogenetic analysis was conducted by using MEGA software version 4.0 (*10*). Complete nucleotide sequence of porcine kobuvirus (strain *Kobuvirus*/swine/S-1-HUN/2007/Hungary) was submitted to GenBank under accession no. EU787450.

Two (13.3%) of 15 samples were positive for porcine sapoviruses; however, a consequent nonspecific, ≈1,100-nt, strong, and single PCR product was found in all samples by agarose gel electrophoresis (data not shown). The nucleotide sequence of the 1,065-nt nonspecific PCR product was determined. Genetic and amino acid similarity was found to be bovine (U-1) and human Aichi kobuvirus 3C (87 nt) and 3D (978 nt) regions in GenBank database by using BLAST (http://blast.ncbi.nlm.nih.gov/Blast.cgi). Nucleotide and amino acid identity of the 3C–3D regions were 73%–79% and 69%–70% to U-1 strain and Aichi virus, respectively. The phylogenetic tree confirmed that S-1-HUN belonged to kobuviruses and formed a distinct lineage (Figure). Cleavage sites for 3C and 3D was Q/S. Highly conserved amino acid motif KDELR in 3D (RNA-dependent RNA polymerase) region and high rate of cytidine (29%) and uracil (26%) nucleoside composition were seen in the 3C and 3D parts of the genome of strain S-1-HUN; both are suspected to be a typical skew of kobuviruses (*3*).

**Figure:**
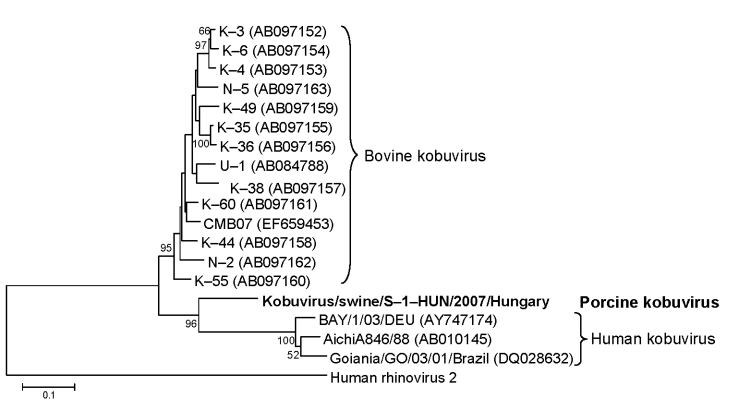
Phylogenetic tree of porcine kobuvirus (Kobuvirus/swine/S-1-HUN/2007/Hungary, GenBank accession no. EU787450), based upon the 1,065-nt fragment of the kobuvirus 3C/3D regions. The phylogenetic tree was constructed by using the neighbor-joining clustering method; distance was calculated by using the maximum composite likelihood correction for evolutionary rate with help of the MEGA version 4.0 software (*10*). Bootstrap values (based on 1,000 replicates) for each node if >50% are given. Reference strains were obtained from GenBank. The human rhinovirus 2 strain (X02316) was included in the tree as an outgroup. Scale bar indicates nucleotide substitutions per site.

Most picornavirus genera consist of >2 species (*1*). Our study reports detection of kobuvirus in domestic pigs. Serendipitously, the generic calicivirus primers p289 and p290, designed for a calicivirus RNA-dependent RNA polymerase region, amplified a kobuvirus 3C/3D region when specimens were tested for porcine caliciviruses by RT-PCR. Comparison of the primers p289 and p290 and the S-1-HUN sequence showed that there are 12- and 11-bp homologous regions between the kobuvirus and the 3′ end of the primer sequences, respectively. Reverse primer p289 designed for calicivirus (norovirus and sapovirus) conserved amino acid 3D motif YGDD, which is also present in kobuviruses.

All apparently healthy animals <10 days of age carried the kobuvirus, which was excreted in the feces. These results indicate a general circulation and endemic infection of kobuvirus on the tested farm. In addition, because of its analogy to other picornaviruses and because bovine kobuvirus was first detected in culture medium that originated from cattle sera (*1**,**3*), we cannot exclude the possibility that the S-1-HUN–like kobuvirus can cause viremia (and generalized infection) in swine. S-1-HUN–like virus may typically cause asymptomatic infections in swine. However, epidemiologic and molecular studies are required regarding the importance of this virus as a causative agent of some diseases of domestic pigs and related animals. Sequence analysis of the complete nucleotide and amino acid sequences of coding (L, P1, P2, and P3: 7,467 nt) and noncoding regions and the genetic organization strain indicate that S-1-HUN is a typical kobuvirus. Phylogenetic analysis shows that S-1-HUN strain is genetically included in the genus *Kobuvirus* but is distinct from Aichi and bovine kobuviruses. Porcine kobuvirus strain S-1-HUN is a candidate for a new, third species of the genus *Kobuvirus*.
